# Differential generation of parasite-specific Th2 and T follicular helper cells distinguishes resistant and susceptible mouse strains

**DOI:** 10.3389/fimmu.2026.1830307

**Published:** 2026-07-01

**Authors:** Hongwei Zhang, Joshua Adjah, Bhavya Kapse, Nicole Affinass, Friederike Ebner, Susanne Hartmann, Sebastian Rausch

**Affiliations:** Institute of Immunology, Department of Veterinary Medicine, Freie Universität Berlin, Berlin, Germany

**Keywords:** B cell, follicular T helper cell, gastrointestinal nematode, *Heligmosomoides polygyrus bakeri*, IgG1, parasite-specific Th2 cell, regulatory T cell, resistance

## Abstract

**Background:**

Both T helper 2 (Th2) and B cell responses are crucial for controlling gastrointestinal (GI) nematode infections. However, adaptive immune determinants of host susceptibility to GI nematode infection are still not fully understood.

**Methods:**

Adaptive immune responses against the small intestinal nematode *Heligmosomoides polygyrus bakeri* were compared between susceptible C57BL/6 and resistant BALB/c mice. Parasite-specific Th2 responses were quantified via combined detection of CD40-L and type 2 cytokine expression in CD4+GATA-3^high^ cells stimulated with parasite products. Follicular T helper cell (TFH) and germinal center B cell responses were determined in parallel. Potential effects of the different Treg activity evident early during infection were tested by IL-2-induced Treg expansion in C57BL/6 and transient Treg depletion in BALB/c DEREG mice, respectively.

**Results:**

Both mouse lines generated comparable Th2 responses, but GATA-3+ Th2 cells comprised significantly higher proportions of parasite-specific cells in the more resistant BALB/c line. In addition, BALB/c mice exhibited stronger TFH responses and more rapid differentiation of IgG1+ germinal center B cells. The superior quality of the adaptive responses was accompanied by the early expansion of Foxp3+ Treg in BALB/c mice. While B cell transfers conveyed partial resistance to primary infection in the BALB/c line, transient Treg depletion at the onset of infection neither altered the quality of the Th2 effector response nor the extent of TFH and IgG1+ B cell responses, nor the control of parasite fitness. In contrast, Th2 responses were repressed following IL-2-induced Treg expansion in C57BL/6 mice, including the further marginalization of parasite-specific responses.

**Conclusion:**

Together, these results indicate that resistance to GI nematodes depends on the quality rather than the quantity of T helper cell responses.

## 1 Introduction

Infections with gastrointestinal (GI) nematodes are among the most common neglected tropical diseases, affecting an estimated 24% of the global population (1). Resistance to nematode infection varies across human populations and in different breeds of farm animals (2). Similarly, inbred mouse lines exhibit varying susceptibility to infection by GI model nematodes. Infection with the natural mouse parasite *Heligmosomoides polygyrus bakeri*, for example, provides a system for investigations of the immune parameters associated with differential genetic resistance to GI nematode infections. *H. p. bakeri* is closely related to economically important GI nematodes, which are widespread in ruminants worldwide (3, 4). The strictly enteric infection is acquired by oral ingestion of the third larval stage (L3), which invades the submucosa of the upper small intestine where it develops into the L4 and from where the adult worms emerge at day eight after infection. Egg deposition starts shortly after and may continue for up to 9 months (5, 6). Despite differences in life cycles and feeding habits, it is the long persistence of adult worms and their phylogenetic proximity to human pathogenic worm species that make *H. p. bakeri* a model for long-lasting GI nematode infections in humans.

All common inbred mouse lines develop patent infections with *H. p. bakeri* (6), but the time required to control for primary infection varies significantly between mouse lines. Resistant lines, such as BALB/c and SJL, terminate the infection by expelling the adult worms within a few weeks, whereas susceptible lines, such as C57BL/6 mice, remain infected for several months (7). Type 2 immune responses, orchestrated by CD4+GATA-3+ T helper 2 (Th2) cells providing IL-4 and IL-13, are critical for the control of GI nematodes (8). However, previous work performed by our group and others indicated that only a small portion of overall Th2 cells induced in *H. p. bakeri* infection were parasite-specific Th2 cells, the latter identified based on the expression of CD154 (CD40-L) and IL-4 or IL-13 (9–11). CD40-L is a type II transmembrane protein expressed on active T cells and reported to be a reliable cell marker to classify or sort pathogen-specific T cells during infections (9, 12). Resistance to *H. p. bakeri* infection is further associated with the extensive accumulation of innate and adaptive immune cells forming granuloma around the tissue-resident larval stages (5). These inflammatory foci are biased for the accumulation of IL-4Ra+ M2 macrophages in BALB/c and SJL mice, conveying partial resistance to primary infection (5).

Type 2 cytokine-mediated resistance to *H. p. bakeri* infection is counterbalanced by the availability of the type 1 effector cytokine IFN-γ (5, 13). As an extreme case, mice pre-exposed to infection with the type 1 pathogen *Toxoplasma gondii* fail in developing a Th2 response to subsequent infection with *H. p. bakeri* (14). Importantly, type 2-dependent resistance is also subject to counter-balance by the rise of IFN-γ competence in CD4+ and CD8+ T cells along aging, as well as by the genotype dependent bias for Th1-like virtual memory cell formation at steady state (15). In consequence, young C57BL/6 mice exhibit a bias for Th2/1 hybrid cell generation at the expense of classical Th2 effector cell differentiation, which is shared with older BALB/c mice exhibiting a similar bias for Th2/1 hybrid responses, paired with declining resistance to primary infection (15).

Further underlining the importance of the local type-2 immune response for the early control of parasite fitness, we recently linked differences in the metabolic activity of migratory, gut derived dendritic cells with the more extensive expression of the chemokine receptor CCR9 in BALB/c- compared to C57BL/6-derived Th2 cells, providing an explanation for the rapid accumulation of BALB/c Th2 cells at the site of *H. p. bakeri* infection (16). Hence, the pace of Th2 effector cell recruitment as well as the relative proportions of classical Th2 versus Th2/1 hybrid cells constitute parameters decisive for the timely control of parasite fitness and reproduction.

In the current study, we focused on potential differences in the quality of the adaptive responses elicited by *H. p. bakeri* infection in susceptible C57BL/6 and resistant BALB/c mice. We show that resistance correlates with higher proportions of parasite-specific Th2 cells, more robust follicular T helper cell and germinal center B cell responses, as well as with an early expansion of Foxp3+ regulatory T cells in BALB/c compared to C57BL/6 mice. The transfer of B cells isolated from primary infection conferred partial protection against primary infection in BALB/c mice, but not in C57BL/6 mice. The transient depletion of Foxp3+ Treg at the onset of infection neither impacted the relative proportion of parasite-specific Th2 cells nor the extent of TFH and IgG1-bound germinal center responses in BALB/c mice. In contrast, boosting Treg expansion in the susceptible C57BL/6 line resulted in impaired overall Th2 differentiation and a further decline in the proportion of parasite-specific Th2 cells.

## 2 Materials and methods

### 2.1 Mice, parasites, and infection

The experiments performed followed the National Animal Protection Guidelines and were approved by the Berlin Animal Ethics Committee for the protection of animals (LAGeSo, license G0176/16, G0207/19 & G0176/20). Female C57BL/6 and BALB/c mice were purchased from Janvier (Saint Berthevin, France) and kept under specific pathogen-free conditions at the Institute of Immunology, Center for Infection Medicine, Department of Veterinary Medicine, Freie Universität Berlin. BALB/c DEREG mice were a kind gift of T. Sparwasser and bred at the Institute of Immunology. Diphtheria toxin (DT) was administered intraperitoneally (i.p.) to Foxp3^GFP^-negative and -positive littermates at a dose of 1 μg per animal for BALB/c DEREG mice at the day of infection. For IL-2/anti-IL-2 complex (IL-2c) administration, C57BL/6 mice received 2.5 μg IL-2 (eBioscience) with 25 μg anti-IL-2 (clone JES6-1A12; eBioscience) i.p. The two compounds were incubated at room temperature for 30 min and administered immediately before infection. (C57BL/6 × BALB/c) F1 mice infected for 14 days were included in one infection experiment. Mice aged 8–10 weeks were infected by oral gavage with 200 *H. p. bakeri* L3 larvae in drinking water and sacrificed at the designated time points by isoflurane inhalation.

### 2.2 Parasitological parameters

Adult worms were isolated from the small intestine and counted. Individual female worms were cultured in RPMI with 200 U/ml penicillin and 200 μg/ml streptomycin (all from PAA, Pasching, Austria) at 37 °C, 5% CO_2_ for 24 hours on 96 well round-bottom plates. Fecundity was determined by counting the eggs shed per female worm using a binocular microscope and expressed as mean values of 8 worms per mouse. Fecal egg counts were determined by microscopy after flotation in saturated salt solution (NaCl) using McMaster chambers.

### 2.3 Preparation of antigens

*H. p. bakeri* adult worms isolated at day 14 p.i. and 4^th^ stage larvae collected from the intestinal tissue at day 6 p.i. were washed extensively before incubation in RPMI medium containing 100 U/ml penicillin and 100 μg/ml streptomycin. The culture medium was discarded after 24 hours and the worms were washed again before replating in fresh medium. Subsequently, culture supernatants were collected at 2–3 days intervals and replaced with fresh medium over a period of 1–2 weeks. Pooled supernatants were concentrated and diafiltrated into PBS over a 3,000 MWCO Amicon membrane to prepare excretory/secretory *H. p. bakeri* products (HES). Extracts generated by mechanical homogenization of the adult or L4 stage were centrifuged at 10.000 x g for 20 min and the supernatants were passed through a 0.22-μm filter (Schleicher & Schuell, Germany) for sterilization. The protein content of HES preparations and worm extracts was determined by the Pierce BCA protein assay (Thermo Fisher) and the material was stored at −80 °C until applied for restimulation.

### 2.4 Preparation of single cell suspensions

Spleen and mesenteric lymph nodes (MLN) were processed into single cell suspensions by forcing the organs through 70 μm cell strainers (BD Bioscience, San Jose, CA). Erythrocytes were removed from spleen samples using ice-cold ACK lysis solution for 3 min. The cells were then washed and re-suspended in cRPMI (100 U/ml penicillin, 100 μg/ml streptomycin, 5% fetal calf serum). Cells from the small intestinal lamina propria (siLP) were isolated as described before (14). Cells were counted using a CASY automated cell counter.

### 2.5 Generation and antigen loading of bone marrow-derived dendritic cells

Bone marrow cells were isolated from the tibia and femur of both hind legs after clearing excess tissue from the bones using sterile cotton gauges. Under the sterile bench, the bones were briefly washed in 70% EtOH followed by washing with RPMI. The bones were then opened from both ends and flushed into a clean Petri dish with RPMI (100 U/ml penicillin, 100 μg/ml streptomycin, 1% fetal calve serum) using a 27G needle. The bone marrow was dissociated by vigorous pipetting and filtered through 70 μm cell strainers, washed once with RPMI, followed by incubation in 1 ml ice cold ACK lysis solution for 3 min. The cells were washed one more time, resuspended in cRPMI medium and counted using a CASY automated cell counter. 5–6 x 10^6^/ml were cultured in cRPMI medium with 20 ng/mL GM-CSF at 37 °C, 5% CO_2_. On day 3, an equal volume of fresh medium with 20 ng/mL GM-CSF was added. On day 6, BmDC were counted, seeded at 2 x 10^5^ cells/well in a 96 well round-bottom plate and incubated with HES or worm extracts overnight.

### 2.6 T cell stimulation

To determine parasite-specific CD4+ T cell responses, 2x10^6^ cells derived from MLN, spleen or siLP of naïve control or *H. p. bakeri* infected mice were added to bmDC that had been preincubated with HES or worm extracts (both applied at 10-25 µg/ml) as described above or were left unloaded (control DC). After 2 hours of DC/T cell interaction, brefeldin A (3 µg/ml, ThermoFisher) was added for another 6-12 hours. Further conditions included the stimulation with 1 μg/ml phorbol 12-myristate 13-acetate (PMA) and 1 μg/ml ionomycin (both from Sigma-Aldrich), anti-CD3/anti-CD28 antibodies (2 µg/ml each; clones: 145-2C11 and 37.51, both from Biolegend) or *Staphylococcus* enterotoxin B (SEB, 1 µg/ml, Sigma-Aldrich) for 30 min at 37 °C and 5% CO_2_ followed by the addition of brefeldin A and incubation for another 4 hours. Samples incubated with control DC served to adjust the gating of CD40-L^high^ parasite-specific cells in the respective stimulated samples.

### 2.7 Th2 cell sorting

Cells were sorted according to the “Guidelines for the use of flow cytometry and cell sorting in immunological studies” (17). CD4+ T cells were enriched from pooled spleen/mesenteric lymph nodes using the untouched CD4+ T-cell isolation kit (Miltenyi Biotech, Bergisch Gladbach, Germany), followed by sorting of ST2+CD25-CD103- cells on a FACS Aria II sorter. 2.5 x 10^4^ CD4+ST-2+ cells were mixed with 2 x 10^4^ bmDC that had been pre-incubated with HES as described above. Culture supernatants were collected after 96 hours and used for cytokine quantification by ELISA.

### 2.8 B cell sorting and transfer

B220+ B cells were purified to >95% from spleen and MLN of day 14 infected donor mice using the pan B cell isolation kit II (Miltenyi Biotech). 2x10^7^ B220+ B cells sorted from *H. p. bakeri* infected C57BL/6 or BALB/c donors were transferred intravenously to respective naïve recipients which were subsequently infected with 200 *H. p. bakeri* L3 larvae. Parasitological parameters were determined 2 weeks later.

### 2.9 Cytokine detection by ELISA

Supernatants were analyzed for IL-4, IL-5, IL-13 and IL-10 using Ready-Set-Go Elisa Kits (eBioscience, San Diego, CA, USA) according to the manufacturer’s instructions.

### 2.10 Flow cytometry

2x10^6^ cells were transferred to conical 96-well plates and surface markers were stained for 10min on ice in PBS with 1% FCS, including a fixable viability dye (eFluor 506 or eFluor 780; ThermoFisher, Waltham, USA). CXCR5 and PD-1 were stained in culture (cRPMI) at 37 °C, 5% CO_2_ for 30 min. For intracellular (ic) detection of transcription factors, cytokines, and CD40-L, stimulated cells were fixed (30 min at room temperature or overnight at 8 °C), permeabilized (2 washing steps of 5 min each), and stained (30 min on ice), using Fix/perm and Perm/wash buffers (ThermoFisher). Purified anti-mouse CD16/CD32 (FcγRII/III, clone 93) was added at 20 µg/ml in surface as well as ic staining steps. Antibodies used from BioLegend: CD4 (RM4-5), CD4 (RM4-4), CD25 (PC61), B220 (RA3-6B2), GL-7 (GL7), IgG1 (RMG1-1), IL-4 (BVD6-24G2), ST-2 (DIH9), streptavidin. From eBioscience/ThermoFisher: CD25 (PC61.5), GATA-3 (TWAJ), T-bet (Bio4B10), CD40-L (MR1), Foxp3 (FJK-16s), IL-5 (TRFK5), IL-13 (eBio13A), CD103 (2E7), PD-1 (J43), streptavidin-conjugates. From BD Bioscience: CXCR5 (2G8). Cells were acquired and analyzed on a FACS Canto II flow cytometer or FACS Aria II sorter (both BD Bioscience, Heidelberg, Germany). FlowJo software 10.2 (Tree Star Inc., Ashland, USA) was used for analysis.

### 2.11 Statistical analysis

Statistical analysis was performed using GraphPad Prism software (La Jolla, CA, USA). Results are displayed as mean values ± SD or in some cases displayed as mean values + SD to make the figure more concise, significance is displayed as *p<0.05, **p<0.01, ***p<0.001, ****p<0.0001. Results were tested for normal distribution using the Shapiro-Wilk normality tests, followed by ANOVA or Kruskal-Wallis combined with Tukey’s or Dunn’s multiple comparison testing.

## 3 Results

### 3.1 Delayed control of nematode infection in susceptible C57BL/6 mice despite robust Th2 cell generation

**Figure 1:**
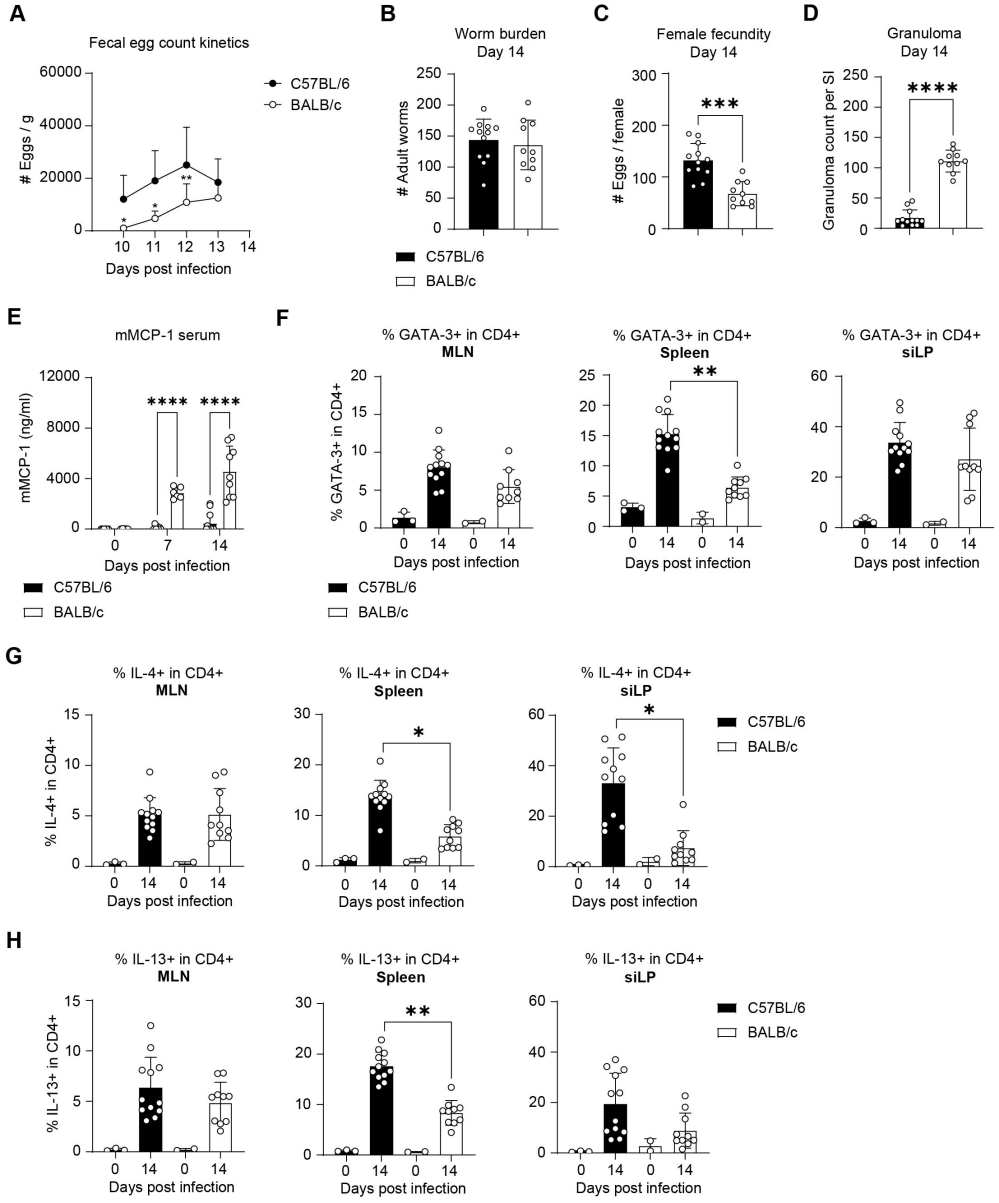
Differential nematode fecundity despite similar Th2 responses in *H. p. bakeri*-infected C57BL/6 mice and BALB/c mice. Age-matched female C57BL/6 and BALB/c mice were infected with 200 *H. p. bakeri* larvae and dissected on day 14 post-infection (p.i.), next to uninfected controls. **(A)** Kinetics of fecal egg counts. **(B)** Worm burden. **(C)** Female worm fecundity. **(D)** Granuloma numbers. **(E)** Serum levels of mouse mast cell protease-1 (mMCP-1) were measured by ELISA at baseline (day 0) and days 7 and 14 post-infection. **(F–H)** Flow cytometric analysis of CD4^+^ T cell responses: frequencies of **(F)** GATA-3^+^, **(G)** IL-4^+^, and **(H)** IL-13^+^ cells within the CD4^+^ T cell population in the mesenteric lymph nodes (MLN), spleen, and small intestinal lamina propria (siLP) at days 0 and 14 post-infection. Data are pooled from 3–4 independent experiments, with n=3–5 mice per group. Statistically significant differences are indicated; *P < 0.05, **P < 0.01; ***P < 0.001; ****P < 0.0001, Mann-Whitney test, Kruskal-Wallis test, or two-way ANOVA test.

To define determinants of host resistance to intestinal nematode infection, we compared the immune responses elicited by *H. p. bakeri* in susceptible C57BL/6 and more resistant BALB/c mice. In accordance with previous studies (5, 15), BALB/c mice released significantly lower numbers of nematode eggs in the 2^nd^ week of infection compared to C57BL/6 mice (**Figure 1A**). Decreased egg shedding was not due to an early difference in worm burdens (**Figure 1B**), but the result of significantly lower fecundity of individual BALB/c-derived female worms (**Figure 1C**). Consistent with previous work (5, 15), numerous macroscopic granulomas were observed in the small intestinal wall of the more resistant BALB/c line (**Figure 1D**). BALB/c mice also exhibited significantly higher serum levels of mucosal mast cell protease-1 (mMCP-1), indicative of extensive mast cell activity at the site of infection (**Figure 1E**). Surprisingly, however, Th2 effector cells marked by high expression of GATA-3 were just as prominent in the mesenteric lymph nodes (MLN) and small intestinal tissue of C57BL/6 mice as in BALB/c mice on day 14 after infection, and the proportion of Th2 cells in the spleen was significantly higher in C57BL/6 than in BALB/c mice (**Figure 1F**). A higher proportion of CD4+ T cells in the MLN of BALB/c compared to C57BL/6 mice (32.59+/-3.23% vs. 22.44+/-3.10%; *P* = 0.0018) translated to a significantly higher absolute number of Th2 cells in MLN of BALB/c mice at day 14 post infection (**Supplementary Figure 1A**). However, similar absolute numbers of Th2 cells were present in spleen and small intestine of the two mouse lines at day 14 p.i. (**Supplementary Figure 1A**). The evaluation of type 2 cytokine expression in CD4+ T cells confirmed the finding of similar or stronger Th2 responses in C57BL/6 compared to BALB/c mice (**Figures 1G, H**; **Supplementary Figure 1B**). Hence, extensive local and systemic Th2 responses translated to high resistance in BALB/c, but not in C57BL/6 mice.

### 3.2 Th2 cells in highly susceptible C57BL/6 mice comprise fewer parasite-specific cells

**Figure 2:**
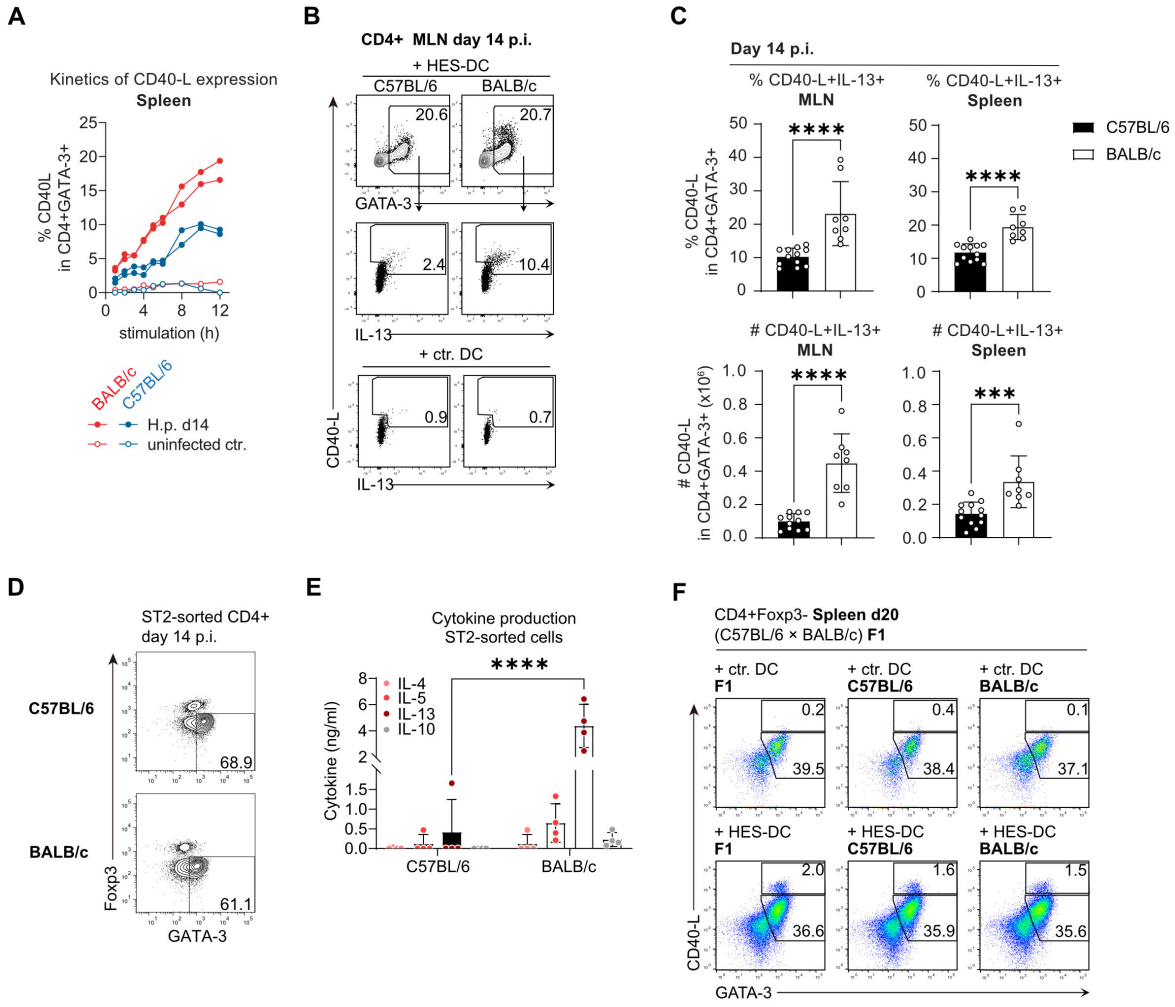
Higher proportions of parasite-specific Th2 cells in *H. p. bakeri*-infected BALB/c compared to C57BL/6 mice. **(A)** Kinetics of CD40-L upregulation in GATA-3+ cells isolated from the spleen of day 14 infected C57BL/6 and BALB/c mice upon exposure to dendritic cells loaded with HES. One out of two experiments with similar results, each performed with 2 mice per mouse strain, is shown. **(B)** Representative plots depicting the gating of CD40-L+IL-13+ parasite-specific Th2 cells. Top row: CD4+ T cells isolated from MLN of C57BL/6 and BALB/c mice following stimulation with HES-DC. 2^nd^ row: Gating of parasite-specific GATA-3+ cells marked by CD40-L and IL-13 expression. 3^rd^ row: Control samples cultured with unloaded DC (ctr. DC) and used to adjust the gating of CD40-L and IL-13 signals. **(C)** Proportions of parasite-specific CD40-L+IL-13+ cells within GATA-3+ cells and absolute numbers of parasite-specific cells in MLN and spleen on day 14 post-infection. Data are pooled from 2–3 experiments, each performed with 3–4 mice. **(D)** Enrichment of GATA-3+ Th2 cells in ST-2-sorted CD4+ T cells on day 14 post-infection. **(E)** ELISA-detection of cytokine production by ST-2-sorted cells after co-culture with HES-DC for 96 hours. Data derived from one experiment with 4 mice per background. **(F)** Plots depicting CD40-L expression by CD4+ T cells derived from the spleen of a 20-day infected C57BL/6 x BALB/c F1 mouse after culture with unloaded DC (ctr. DC) or HES-loaded DC generated from F1, C57BL/6, and BALB/c mice. Representative data from n=3 F1 mice are shown. Statistically significant differences are indicated; ***P < 0.001; ****P < 0.0001, Mann-Whitney test, Kruskal-Wallis test, or two-way ANOVA test.

The different resistance of C57BL/6 and BALB/c mice to *H. p. bakeri* infection despite the generation of comparably strong Th2 responses raised the question of whether the quality of the T cell response differed between the two mouse lines. Protection in adaptive immunity is typically tied to particular antigens/epitopes, and both host and pathogen genotypes determine which antigens are expressed, presented, and recognized. To see if the proportions of parasite-specific cells differed in the GATA-3+ effector populations generated by infected C57BL/6 and BALB/c mice, we hence stimulated the cells with parasite products and determined the expression of CD40-L, which allows the quantitative analyses of antigen-specific CD4+ T cells (9, 12). Bone marrow-derived dendritic cells loaded with the excretory/secretory products of *H. p. bakeri* (HES-DC) were used as antigen-presenting cells. A comparison of CD40-L upregulation in spleen cells isolated from the two mouse lines at day 14 post-infection and stimulated with HES-DC for up to 12 hours showed that BALB/c-derived Th2 cells consistently comprised higher proportions of HES-specific cells marked by CD40-L expression (**Figure 2A**). The combined detection of CD40-L with IL-4 or IL-13 production confirmed higher proportions as well as absolute numbers of parasite-specific Th2 cells in MLN and spleen of BALB/c compared to C57BL/6 mice at day 14 and 7 post-infection (**Figures 2B, C**; **Supplementary Figure 2**). To validate this finding, we sorted CD4+ST2+ T cells and cultured these T cell pool enriched for Th2 cells from C57BL/6 and BALB/c mice with HES-DC. Consistent with the pattern of CD40-L and IL-13 expression determined by flow cytometry, Th2-enriched cells from BALB/c mice released significantly higher amounts of IL-13 compared to their C57BL/6-derived counterparts when cultured with HES-loaded DC (**Figures 2D, E**).

Resistance to primary *H. p. bakeri* infection was shown to be linked with the MHCII (I-A/I-E) haplotype (6, 18, 19). To rule out differences in antigen uptake/processing between bone marrow-derived DC, we infected C57BL/6 x BALB/c F1 mice and cultured their spleen cells with HES-loaded bmDC generated from F1, C57BL/6 (I-A^b^/I-E^null^) and BALB/c mice (I-A^d^/I-E^d^). As shown in **Figure 2F**, slightly more of the Th2 cells derived from F1 mice on day 20 post-infection responded to HES presented by F1-DC as compared to HES presented by C57BL/6- or BALB/c-derived DC (**Figure 2F**). However, HES presented by DC derived from the parental lines elicited highly similar responses in F1 Th2 cells, indicating similarly effective antigen presentation by DC from C57BL/6 and BALB/c mice. Underlining the sensitivity of the CD40-L readout, CD4+ T cells derived from the parental lines displayed similar frequencies of alloreactive CD4+ T cells responding with CD40-L expression in culture with mismatched DC, irrespective of HES-loading (**Supplementary Figure 3**). Together, these data show that the higher resistance of the BALB/c compared to the C57BL/6 line to *H. p. bakeri* infection is associated with a higher number of parasite-specific cells within the pool of GATA-3+ Th2 effector cells.

### 3.3 Stable proportions of parasite-specific and non-specific Th2 cells along the course of primary infection

**Figure 3:**
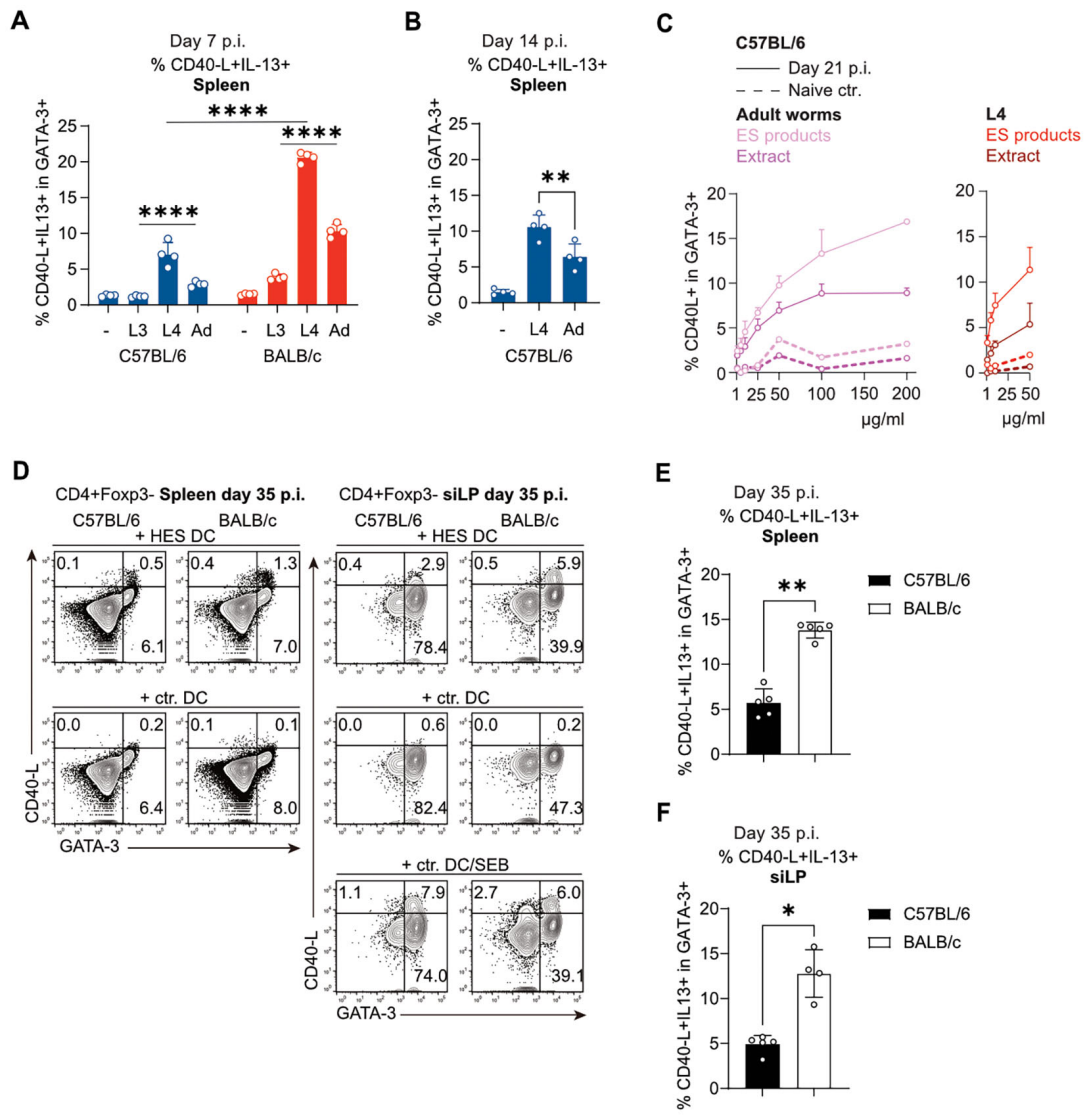
BALB/c mice harbor higher proportions of *H. p. bakeri*-specific cells along the course of primary infection. **(A)** Frequencies of parasite-specific in GATA-3+ Th2 cells from C57BL/6 and BALB/c mice on day 7 post-infection as determined after restimulation with lysates derived from the infective L3, tissue-resident L4, and adult (Ad) worms. **(B)** Frequencies of parasite-specific cells in C57BL/6-derived Th2 cells stimulated with L4 and adult worm lysates on day 14 post-infection **(C)**. Responses to ES products and lysates derived from adult worms (1-200µg/ml) and L4 (1-50µg/ml) were surveyed with spleen cells of day 21 infected C57BL/6 mice. n=3. **(D)** Representative plots depicting HES-specific responses of Th2 cells derived from spleen and siLP of the two mouse lines on day 35 post-primary infection. **(E, F)** Comparison of proportions of HES-specific Th2 cells in spleen and siLP from both mouse lines on day 35 post-primary infection. One experiment performed with n=4 per background. Statistically significant differences are indicated; *P < 0.05; **P < 0.01; ****P < 0.0001, Mann-Whitney test, Kruskal-Wallis, or two-way ANOVA test.

While the proportions of HES-responsive Th2 cells differed clearly between the mouse lines, the proportion of Th2 cells responding to antigens derived from the larval and adult parasite stages was surprisingly low in both BALB/c and C57BL/6 lines. For a more comprehensive evaluation of the parasite-specificity of Th2 cells, we hence compared the responses of GATA-3+ cells of both mouse lines to the products of the infectious third-stage larvae (L3), the tissue-resident L4, and the adult worms (**Figure 3**). On day 7 post-infection, before the transition of the L4 to the adult stage, restimulation of Th2 cells from both mouse lines with L4 products elicited CD40-L upregulation in approximately twice as many Th2 cells compared to the response induced by adult worm products, whereas very few cells responded to extracts derived from the infective L3 stage (**Figure 3A**). Interestingly, on day 14, i. e. just under a week after transition of the L4 to the adult stage, a similar picture emerged, with approximately twice as high a response from Th2 cells after stimulation with L4 antigens compared to the response to antigens from adult worms (**Figure 3B**). Another week later in the infection (d21), we compared the responses to ES and somatic products of both life stages. Expectedly, the responses to ES products were consistently stronger compared to those driven by worm extracts (**Figure 3C**). Furthermore, the bias for reactivity to L4 products was no longer detectable at three weeks post infection, with similar CD40-L expression induced by L4/adult ES and by extract antigens of the two life stages (**Figure 3C**). A comparison of Th2 cells from both mouse lines on day 35 post primary-infection revealed stable proportions of HES-specific and non-responsive cells in the chronic stage of infection, with BALB/c-derived Th2 cells still harboring higher frequencies of HES-responsive cells (**Figures 3D–F**). Hence, Th2 cells generated by the more resistant BALB/c line in response to *H. p. bakeri* infection comprise more parasite-specific cells, whereas both BALB/c and the less resistant C57BL/6 line generate large quantities of Th2 cells neither responding to L4 nor to adult worm products.

### 3.4 The nematode-induced response is biased for the generation of follicular T helper cells and rapid IgG1 class switch in resistant BALB/c mice

**Figure 4:**
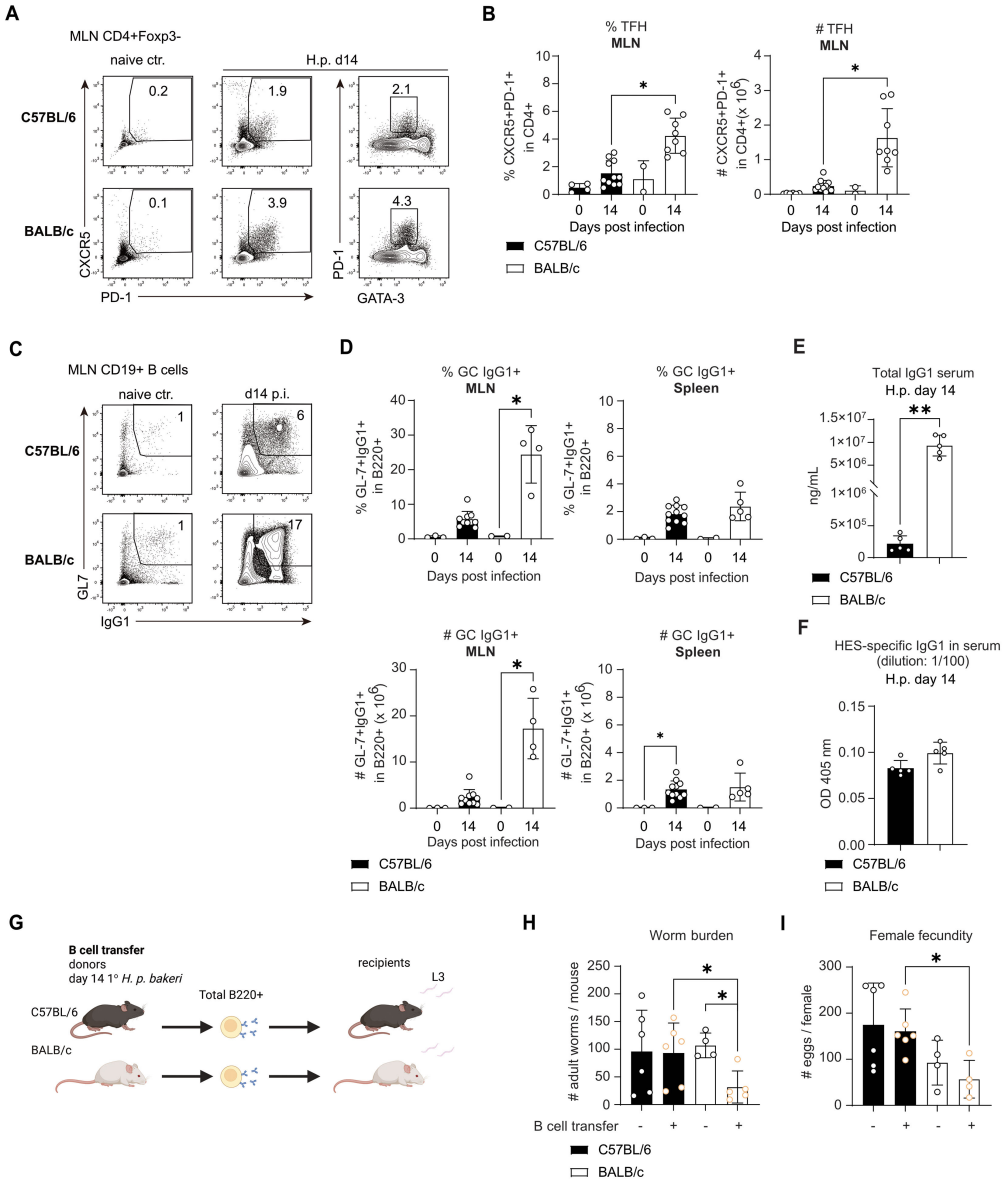
Stronger TFH responses and rapid class switch in *H. p. bakeri*-infected BALB/c mice**. (A)** Representative plots depicting TFH detection in naïve and day 14 infected C57BL/6 and BALB/c mice based on PD-1 and CXCR5 expression (1^st^ and 2^nd^ column) and the moderate GATA-3 signal intensity of PD-1+ TFH compared to GATA-3^high^ Th2 effector cells (3^rd^ column). **(B)** Frequencies and absolute numbers of CXCR5+PD-1 TFH cells in MLN on day 14 post-infection. **(C)** Gating of IgG1 class-switched cells expressing the germinal center marker GL7 in CD19+ B cells derived from MLN of naïve and day 14 infected C57BL/6 and BALB/c mice. **(D)** Frequency and absolute number of germinal center IgG1+ B cells in MLN and spleen on day 14 post-infection. **(E, F)** Total **(E)** and HES-specific IgG1 **(F)** in serum of infected and naïve mice. **(G)** Experimental setup of B cell transfer in C57BL/6 and BALB/c mice. **(H)** Worm burden on day 14 post-infection in C57BL/6 and BALB/c mice, either left untreated or receiving a transfer of B220+ sorted B cells from day 14 infected donors. **(I)** Female worm fecundity in C57BL/6 and BALB/c infected mice 14 days after infection +/- transfer of B cells from day 14 infected donors. Data in B and D-F are pooled from 2–3 independent experiments, each performed with n=2–3 mice per group. Data in H and I derive from 2 experiments with 2–3 mice per group. Statistically significant differences are indicated; *P < 0.05; **P < 0.01, Mann-Whitney test, Kruskal-Wallis test, or two-way ANOVA test.

Next, we asked if the different proportions of parasite-specific CD4+ T cells induced in C57BL/6 versus BALB/c mice in response to *H. p. bakeri* infection were joined with differential T follicular helper cell and B cell responses. Earlier work performed with 4get/KN BALB/c mice (which report IL-4 competence by GFP and IL-4 release by huCD2 surface expression) showed that approximately 40% of the IL-4 competent cells present in the MLN at day 14 post *H. p. bakeri* infection actively express IL-4. These cells were identified as T follicular helper cells (TFH), both by the expression of CXCR5, PD-1, and IL-21 as well as by their preferential localization to the B cell area of the MLN (20). To figure out whether the different quality of Th2 effector responses in BALB/c and C57BL/6 mice was accompanied by discrete patterns of TFH differentiation, we determined the proportion of TFH cells based on surface expression of PD1 and CXCR5. Indeed, the frequencies and absolute numbers of PD-1+CXCR5+ TFH cells were significantly higher in MLN of BALB/c mice at day 14 post-infection compared to C57BL/6 mice (**Figures 4A, B**). PD1+ TFH cells formed a distinct population, marked by intermediate GATA-3 expression from GATA-3^hi^ Th2 cells (**Figure 4A**).

The more robust generation of TFH cells was accompanied by far higher frequencies and absolute numbers of IgG1+ CD19+ B cells expressing the germinal center markers GL7 and PNA (**Figures 4C, D**, and data not shown). Accordingly, IgG1 levels in serum were remarkably higher in BALB/c mice on day 14 of primary infection (**Figure 4E**), while a trend for higher HES-specific IgG1 in sera did not reach significance compared to C57BL/6 mice (**Figure 4F**). To see whether the more rapid B cell response contributed to the higher resistance of BALB/c mice to primary nematode infection, we compared the two mouse lines for the effects of adoptive B cell transfers on worm burden and egg production (**Figure 4G**). B220+ B cells isolated from BALB/c donors on day 14 post infection and transferred to naïve recipients conveyed more robust resistance to primary *H. p. bakeri* infection, evident in the lower worm burdens and reduced fecundity of female worms two weeks post infection (**Figures 4H, I**). In contrast, neither worm burden nor female worm fecundity differed from control animals in C57BL/6 mice upon B cell transfer (**Figures 4H, I**). Tagen together, BALB/c mice exhibit a bias for TFH differentiation and rapid IgG1 germinal center responses, resulting in higher total IgG1 levels in primary *H. bakeri* infection. Despite similar parasite-specific IgG1 responses in the two mouse lines, partial protection was only seen transferred with B cells from primary infected BALB/c, but not C57BL/6 mice.

### 3.5 Divergent outcome of Treg manipulation in BALB/c and C57BL/6 mice

**Figure 5:**
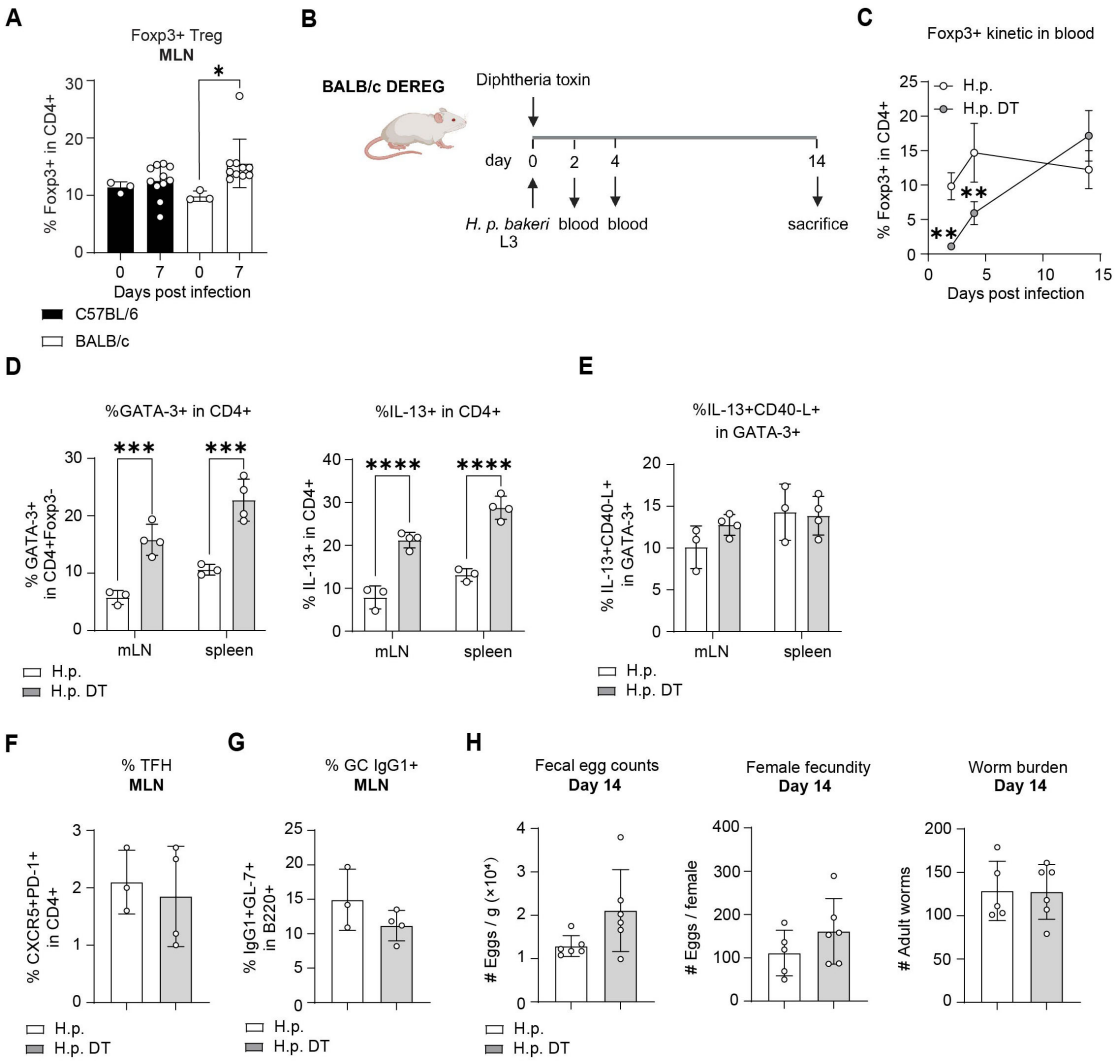
Transient Treg depletion in BALB/c DEREG mice leads to elevated Th2, along with stable TFH and IgG1 responses. **(A)** Frequencies of Foxp3+ Treg cells in MLN of naïve and day 14 infected C57BL/6 and BALB/c mice. Data are pooled from 3 experiments, each performed with 3–4 infected mice per background. **(B)** Experimental setup. BALB/c DEREG mice received a single dose of diphtheria toxin (DT) immediately before *H. p. bakeri* infection. Treg depletion was monitored in the blood, and mice were dissected on day 14 post-infection. **(C)** Foxp3+ expression in peripheral blood CD4+ cells on day 2, 4, and 14. **(D)** Frequencies of Th2 cells in MLN-derived CD4+ T cells according to GATA-3+ and PMA/ionomycin-induced IL-13+ expression. **(E)** Frequencies of CD40-L+ parasite-specific cells in the GATA-3+ Th2 population derived from MLN and spleen as determined following stimulation with HES-DC. **(F)** Frequencies of TFH cells in MLN CD4+ T cells. **(G)** Frequencies of GL7+IgG1+ cells in MLN-derived B220+ B cells on day 14 of infection. **(H)** Fecal egg counts, female worm fecundity, and intestinal worm burdens on day 14 of infection. Data are pooled from 2 independent experiments, with n=2–3 mice per group. Statistically significant differences are indicated; *P < 0.05, **P < 0.01; ***P < 0.001; ****P < 0.0001, Mann-Whitney test or Kruskal-Wallis test.

Earlier work of our and other groups demonstrated a strong increase in Treg activity and suppressive capacity in *H. p. bakeri*-infected BALB/c mice compared to more subtle changes in infected C57BL/6 mice (5, 21, 22). In accordance with earlier studies, BALB/c, but not C57BL/6 mice displayed a significant expansion of Foxp3+ Treg cells in MLN at day 7 post infection (**Figure 5A**). To see if stricter control of the T cell response by Foxp3+ Tregs conferred an advantage in terms of the quality of CD4+ T cell responses, we temporarily reduced Foxp3+ Tregs at the onset of *H. p. bakeri* infection in BALB/c DEREG mice (**Figures 5B, C**). Transient depletion of Foxp3+ Treg translated to generally increased Th2 responses, evident in significantly higher frequencies of GATA-3^high^ effector cells and more prominent IL-13 and IL-4 production in MLN and spleen (**Figure 5D**; **Supplementary Figure 4A**). However, the proportions of parasite-specific Th2 cells as well as TFH and IgG1+ GC B cell responses and the number of small intestinal granuloma were similar in the two groups at day 14 post-infection (**Figures 5E–G**; **Supplementary Figure 4B**). Accordingly, both groups displayed similar parasite egg production and comparable worm burdens (**Figure 5H**).

**Figure 6:**
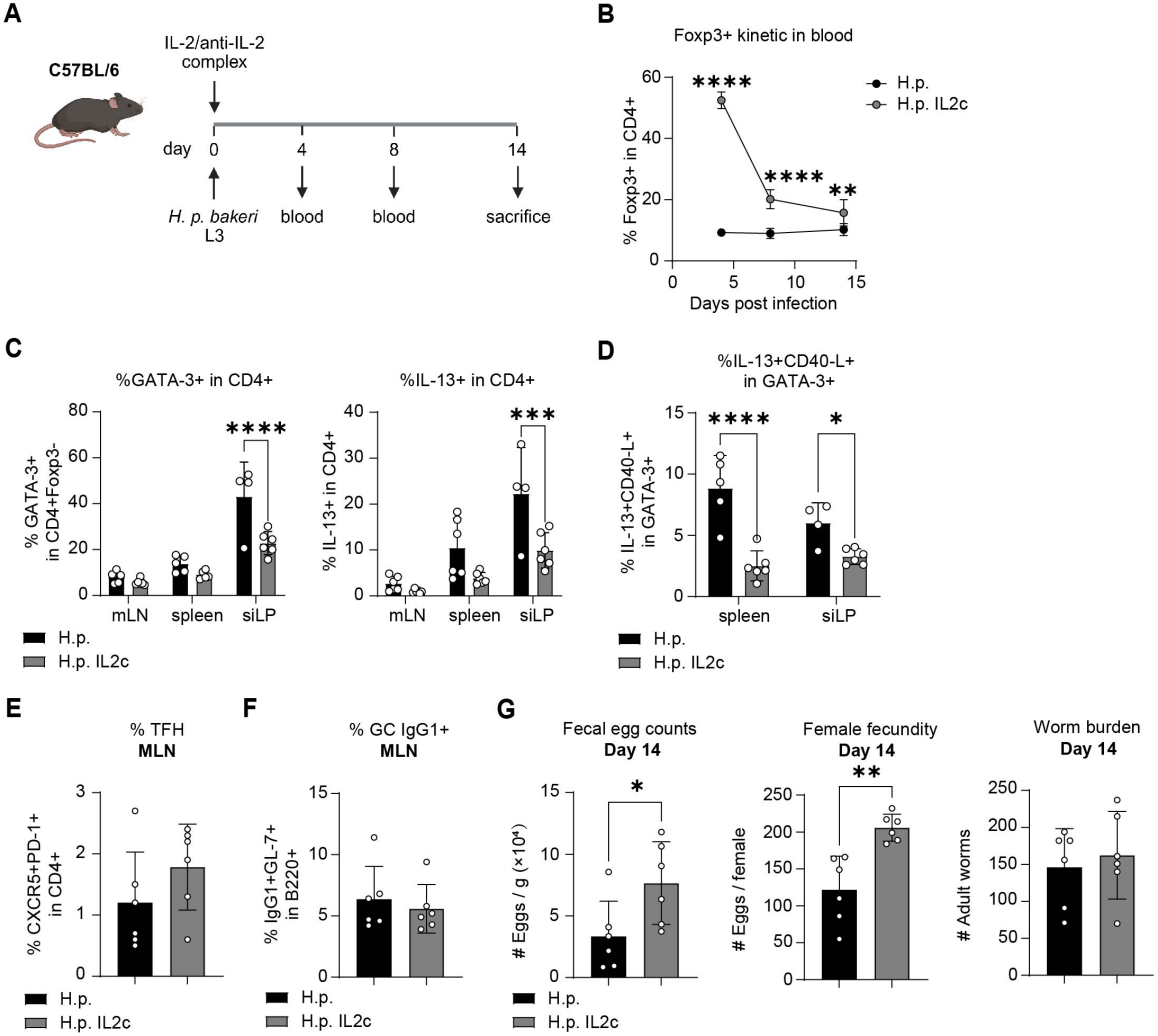
IL-2-induced Treg expansion in C57BL/6 mice selectively impedes the Th2 response, resulting in further decline of resistance. **(A)** C57BL/6 mice received an intraperitoneal application of a recombinant IL2/anti-IL2 antibody complex directly before *H. p. bakeri* infection. **(B)** Frequencies of Foxp3+ Treg in peripheral blood CD4+ cells on day 4, 8, and 14 after infection. **(C)** Frequencies of Th2 cells in MLN-derived CD4+ T cells according to GATA-3+ and PMA/ionomycin-induced IL-13+ expression. **(D)** Frequencies of CD40-L+ parasite-specific cells in the GATA-3+ Th2 population derived from spleen and siLP on day 14 post-infection as determined following stimulation with HES-DC. **(E)** Frequencies of TFH cells in MLN CD4+ T cells. **(F)** Frequencies of GL7+IgG1+ cells in MLN-derived B220+ B cells on day 14 of infection. **(G)** Fecal egg counts, female worm fecundity, and intestinal worm burdens on day 14 of infection. Data are pooled from 2 independent experiments, each performed with n=3 mice per group. Statistically significant differences are indicated; *P < 0.05, **P < 0.01; ***P < 0.001; ****P < 0.0001, Mann-Whitney test or Kruskal-Wallis test.

In a reverse approach, we assessed the consequences of Treg expansion via application of IL-2/anti-IL-2 antibody complex (IL-2c) at the onset of *H. p. bakeri* infection in the more susceptible C57BL/6 line (**Figure 6A**). IL-2c treatment provoked a dramatic, yet transient increase of Foxp3+ Treg (**Figure 6B**) along with impaired Th2 responses at day 14 p.i. (**Figure 6C**; **Supplementary Figure 4C**). Notably, the induced rise of Foxp3+ Treg resulted in a further decline of the proportion of parasite-specific Th2 cells marked by CD40-L and IL-13 and IL-4 expression in response to HES-stimulation in C57BL/6 mice (**Figures 6C, D**; **Supplementary Figure 4D**). While the proportions of TFH cells and of IgG1+ GC B cells were comparable in C57BL/6 mice with boosted Treg responses and the infected control group (**Figures 6E, F**), the more limited overall and parasite-specific Th2 response correlated with a significant rise of fecal egg counts and female worm fecundity and with reduced small intestinal granuloma formation on day 14 post-infection (**Figure 6G**; **Supplementary Figure 4E**).

In summary, these data show that a brief loss of Treg resulted in stronger Th2 responses in the relatively resistant BALB/c line. However, the quality in terms of the proportion of parasite-specific Th2 cells remained unaffected, and the larger number of parasite-specific and non-specific Th2 cells did not lead to improved control of the worm infection. In contrast, the support of early Treg responses in C57BL/6 mice resulted in a further reduction of the parasite-specific Th2 response and higher reproductive parasite fitness in this line.

## 4 Discussion

Several studies have previously shown that BALB/c mice are more resistant to infection with the small intestinal nematode *H. p. bakeri* as compared to C57BL/6 mice (5, 15, 16). However, our previous and current work shows that both mouse lines generate similar Th2 responses based on the quantification of GATA-3+ Th2 cells, the cell type is critical for the control of primary as well as challenge infection. In earlier work, we identified qualitative differences concerning the Th2 effector responses between the two mouse lines, namely the higher proportion of IFN-γ competent Th2/1 hybrid cells in *H. p. bakeri*-infected C57BL/6 compared to BALB/c mice (15). Furthermore, we recently demonstrated the more rapid recruitment of GATA-3+ effector cells to the site of infection, associated with more prominent expression of the small intestinal homing receptor CCR9 in Th2 cells of infected BALB/c mice (16). In the current study, we identified higher proportions of parasite-specific Th2 cells and a bias for TFH generation associated with rapid B cell responses as further qualities associated with resistance of the BALB/c line against *H. p. bakeri* infection. Compared to C57BL/6 mice, consistently more of the Th2 cells generated in BALB/c mice in response to *H. p. bakeri* infection upregulated CD40-L expression and type 2 cytokine expression when stimulated with nematode products. This pattern was stable along the course of primary infection, with the higher numbers of HES-responsive cells present in the more resistant strain on day 14 and 35 post-infection, matched the lower egg release early in infection. These findings matched the lower egg release by BALB/c mice early in infection, as well as the almost complete elimination of adult worms by day 35 post-infection, respectively.

Expectedly, the excretory/secretory products actively released by the larval and adult nematodes elicited CD40-L and cytokine expression by more Th2 cells compared to the somatic antigens present in worm extracts. In accordance with stage-specific profiles of protein expression reported earlier (23), more Th2 cells responded to extracts of the L4 than of the adult worms on day 7 and 14 post-primary as well as on day 5 after challenge infection. This probably indicates robust responses induced by somatic antigens available from 4^th^ stage larvae that failed in completing development to adult worms and were degraded within the host tissue. However, a comparison of CD40-L upregulation induced by either the somatic or ES products of larval and adult worms showed that similar numbers of Th2 cells responded to products of the distinct life stages at later stages of infection. Hence, irrespective of the significantly different numbers of *H. p. bakeri*-specific Th2 cells present in the GATA-3+ T cell populations of C57BL/6 and BALB/c mice, the CD40-L-based assessment of parasite-specificity reported congruent kinetics of stage-specific Th2 responses in the two mouse lines.

Our data indicate that non-specific Th2 cells account for the majority of Th2 cells generated in both the susceptible and resistant lines. This finding contrasts with an earlier study, which investigated the T cell response to *N. brasiliensis*, a nematode closely related to *H. p. bakeri* (4), claiming that essentially all Th2 cells in *N. brasiliensis*-infected mice were parasite-specific (24). However, this conclusion was delineated from the absence of Th2 bystander induction in TCR-transgenic T cells in *N. brasiliensis*-infected mice and not based on evidence for TCR-dependent reactivation of the entire population of Th2 cells by parasite antigens (24). Earlier work investigated the cellular sources of IL-4 production in *H. p. bakeri* infection in 4get/KN2 IL-4 reporter mice. In line with our CD40-L/GATA-3 based readout identifying about 13-15% of parasite-specific cells within the GATA-3+ Th2 population present in the peritoneal cavity of *H. p. bakeri* infected mice (data not shown), the earlier study showed that about 15% of the GFP+ IL-4-competent peritoneal Th2 cells upregulated huCD2 indicating active IL-4 expression when stimulated with *H. p. bakeri* antigens within 24 hours (25). Hence, using different techniques, our data and earlier work strongly suggest that the majority of Th2 cells generated in response to *H. p. bakeri* infection are non-specific bystanders.

CD40/CD40-L interaction is essential for T-B cell cooperation and T cell dependent antibody responses *in vivo* (26, 27). Consequently, gene disruption or antibody-mediated blocking of CD40-L results in impaired B cell expansion and IgG1 responses in mice infected with *H. p. bakeri* or *Trichinella spiralis* (28, 29). Importantly, parasite-specific IL-4/-13 responses were more severely impaired compared to mitogen-induced Th2 cytokine release in *T. spiralis* infected CD40-L-deficient mice, indicating a severe defect in parasite-specific response without ablation of overall Th2 induction (29). Along that line, CD40-L blocking was recently reported to result in increased luminal worm counts early in *H. p. bakeri* infection without impacting overall Th2 differentiation (30). To further support our conclusion regarding the importance of the varying degrees of parasite-specific Th2/TFH responses, future studies need to compare the outcome of antibody-mediated blocking of CD40/CD40-L interactions in *H. p. bakeri*-infected C57BL/6 and BALB/c mice. Provided that both strains continue to elicit similarly strong Th2 bystander responses under CD40-L-blockade, this approach should reveal whether the suppression of parasite-specific Th2 responses negates the greater resistance of the BALB/c strain.

Interestingly, earlier studies showed that defective CD40-L signaling resulted in remarkably impaired mast cell responses in both *H. p. bakeri* as well as *T. spiralis* infection (28, 29). In line with these reports, we find stronger systemic and local parasite-specific Th2 responses paired with remarkably higher mMCP-1 levels in sera of *H. p. bakeri* infected BALB/c mice, indicative of more robust mucosal mast cell activation in the more resistant line. Mast cells are crucial for the induction of type-2 responses to infections with *H. p. bakeri* and *Trichinella spiralis*, evident in the severely impaired Th2 differentiation, parasite-specific type 2 cytokine secretion as well as IgG1/IgE production in mast-cell deficient mice (31–33). In *H. p. bakeri* infection, mast cells regulate the production of tissue cytokines and serve as an early source of IL-33 and IL-25 required for the control of worm burden and parasite fecundity (33, 34). It is hence conceivable that the more extensive parasite-specific T effector responses seen in *H. p. bakeri* infected BALB/c mice provide IL-3, IL-4 and IL-9 eliciting the accumulation of mucosal mast cells. In turn, mast cells may provide IL-25 shown to synergize with IL4Ra signaling in the promotion of innate type 2 effector cells, permitting the expulsion of adult worms in chronically infected BALB/c mice (35, 36). Therefore, further studies are needed to determine whether the extent of mast cell activation depends on the intensity of parasite-specific Th2 responses and whether sustained mast cell activation in the more resistant BALB/c strain leads to earlier expulsion of adult worms.

Although our data demonstrate that the quality of the Th2 response at day 14 correlates with reduced fecundity, we cannot exclude an additional, earlier role for mast cells. The early increase in mMCP−1 (day 7) suggests mucosal mast cell activation prior to the peak of the measured effector Th2 response. Therefore, mast cells may contribute to the initial reduction in egg output, either independently or in parallel with developing T−cell immunity. This possibility is consistent with prior evidence that mast cells can regulate *H. p. bakeri* fecundity and does not contradict our emphasis on T−cell quality (33, 34), but rather highlights a temporally distinct, potentially complementary mechanism. Future experiments assessing earlier time points for mast cell activation, CD4+ T cell priming, and fecundity in mast−cell−deficient mice will be needed to resolve this question.

Unfortunately, due to the incompatibility of the conditions required for reliable intracellular detection of CD40-L and cytokine production with the protocol required for PD-1/CXCR5 detection, we were not successful in quantifying parasite-specificity in TFH cells. However, comparing the frequencies of CD4+ T cells marked by PD-1 and CXCR5 expression in two mouse lines, we determined significantly stronger TFH responses in the resistant BALB/c compared to the C57BL/6 line. This pattern was mirrored in significantly more extensive IgG1 class switching and significantly elevated total IgG1 levels in BALB/c compared to C57BL/6 mice on day 14 after primary infection.

Serum transfers from infected to naïve mice provide varying degrees of protection depending on the number of infections undergone by the donors (37–39). We show that B cells from primary infected BALB/c mice, but not C57BL/6 mice, transfer protection against challenge infection. These findings align with earlier work reporting passive immunization against primary infection by serum transfer from challenged, but not primary infected C57BL/6 mice (40) and with the protection following the transfer of memory B cells remaining after a resolved primary infection in BALB/c mice (41). While the antibody-mediated immunity induced by immunization with ES products of *H. bakeri* is entirely based on parasite-specific IgG1 (42), other work reported IgG2c/FcgRI dependent M2 polarization and larval trapping *in vitro* (43) IFN-γ is the main driver of IgG2a/c class switch and we previously identified Th2/1 hybrid cells as a more prominent source of parasite-specific IFN-γ production in C57BL/6 compared to BALB/c mice (13, 15). We hence asked if the different IFN-γ competence was mirrored in IgG2a/c responses. However, total and HES-specific IgG2a/c levels were similarly low in sera and few IgG2a/c+ B cells were detected in MLN of both mouse lines irrespective of the infection status (data not shown).

Given the importance of HES-specific IgG1 in antibody-mediated immunity, it is important to note that at the time of B-cell isolation from infected mice, the serum of BALB/c mice comprised significantly more total IgG1, but the levels of parasite-specific IgG1 were similar between the two mouse lines. The latter aligns with earlier reports of similar parasite-specific IgG1 production in primary infected BALB/c and C57BL/6 mice (44, 45). It is therefore unlikely that varying levels of parasite-specific IgG1 produced by the transferred B cells were responsible for the effective protection observed following B-cell transfers in BALB/c mice, in contrast to C57BL/6 mice. However, IgG1-mediated immunity acts in concert with IL-4Ra/IL-25-dependent innate effector mechanisms (42) and IgG1 likely neutralizes key ES products employed by the parasite to interfere with these effector responses (46–48). *H. p. bakeri*-specific IgG1 produced by transferred B cells may hence synergize with the stronger early parasite-specific Th2 response reported in our study (d7, see **Figure 3**) and, potentially, more robust early M2 polarization reported for primary infected BALB/c compared to C57BL/6 mice (5). Furthermore, although mast cells were not required for the protection via the high antibody titers induced by HES/alum immunization in C57BL/6 mice (42), the extensive activation of mucosal mast cells described in our study shortly after primary infection in BALB/c mice may have acted synergistically with parasite-specific IgG1 in BALB/c B-cell recipients. Taken together, our data imply that parasite-specific IgG1 provided by B cell transfers promotes protective immunity when combined with stronger early Th2 and innate responses. The antibody responses of the two mouse strains may therefore suppress immunomodulation by ES products to a similar extent; however, IgG1 partially alleviating the action of parasite immunomodulators may confer protective immunity more readily in BALB/c mice displaying stronger parasite-specific Th2 responses and innate type-2 effector mechanisms.

Several recent studies identified the early release of IL-13, IL-4, and IL-9 by type 2 innate lymphoid cells (ILC2) as an important mechanism promoting the generation of TFH cells and the formation of germinal center reactions (49–51). In the present study, we did not perform in-depth analyses of the ILC2 lineage; however, quantification of CD4-GATA-3^high^ ILC2-like cells on day 7/14 post infection confirmed previous reports of higher absolute numbers of ILC2 in MLN of infected compared to naïve mice (33,36 and data not shown) In accordance with an earlier study (5), MLN of infected BALB/c mice comprised higher absolute numbers of ILC2-like cells on day 7 compared to C57BL/6 mice. However, the higher number reflected the higher cell density of the MLN rather than a selective expansion of ILC2, as similar frequencies of CD4-GATA-3^high^ cells were observed in the MLN and siLP of both mouse lines, regardless of infection status (**Supplementary Figure 5A**). Notwithstanding the importance of ILC2s for Th2 differentiation (52), these preliminary data suggest that the more prominent TFH and parasite-specific Th2 effector responses observed in *H. bakeri* infected BALB/c compared to C57BL/6 mice developed independently of marked differences in ILC2 expansion.

As BALB/c mice displayed significantly elevated Treg numbers following infection, we asked whether stricter Treg control resulted in relatively lower bystander activation and a bias for TFH/humoral response in the more resistant BALB/c line. However, while transient depletion of Treg at the onset of *H. p. bakeri* infection resulted in stronger Th2 responses, neither the proportion of non-specific bystanders nor the TFH or B cell responses were altered. Strikingly, stronger overall Th2 responses did not translate to more efficient parasite control, as worm burdens and fecundity were unchanged in BALB/c mice following transient Treg depletion. This finding contrasts with an earlier report of reduced worm burdens in BALB/c mice following early Treg depletion by anti-CD25 antibodies (53). Interestingly, the same study showed that more thorough depletion of Foxp3+ Treg by repeated administration of diphtheria toxin in DEREG mice resulted in increased parasite fecundity and delayed expulsion of adult worms, despite similar Th2 cytokine production in response to HES (53). In the present work, we applied a single dose of DT. The more rapid Treg recovery recorded in our experiments compared to the more long-lived Treg reductions reported in earlier depletion approaches may explain the unchanged parasitological parameters detected in our study.

In a converse approach, early Treg expansion enforced in C57BL/6 mice by IL-2/anti-IL-2 antibody complex resulted in reduced Th2 responses and a further decline of parasite-specific cells within GATA-3+ effector cells. Similar to the stable proportions of TFH and B cell responses following transient Treg depletion in BALB/c mice, enforced Treg expansion left TFH and B cell responses unaltered in C57BL/6 mice. Nevertheless, worm numbers and fecundity were significantly increased following Treg expansion, indicating a further decline of resistance along with the qualitative and quantitative decline of Th2 responses in C57BL/6 mice.

Taken together, our study indicates that resistance to GI nematodes correlates with largely depends on a bias towards parasite-specific Th2, TFH responses and rapid IgG1 class switch, rather than the quantity of Th2 cells.

## Data Availability

The original contributions presented in the study are included in the article/**Supplementary Material**. Further inquiries can be directed to the corresponding author.
