# Supplementary material for: Differential generation of parasite-specific Th2 and T follicular helper cells distinguishes resistant and susceptible mouse strains
Source: Front Immunol. 2026 Jul 1;17:1830307. doi: 10.3389/fimmu.2026.1830307 (PMC13368484; doi:10.3389/fimmu.2026.1830307)
Supplement: Supplementary file 1 [file DataSheet1.docx]

Supplementary Material

**
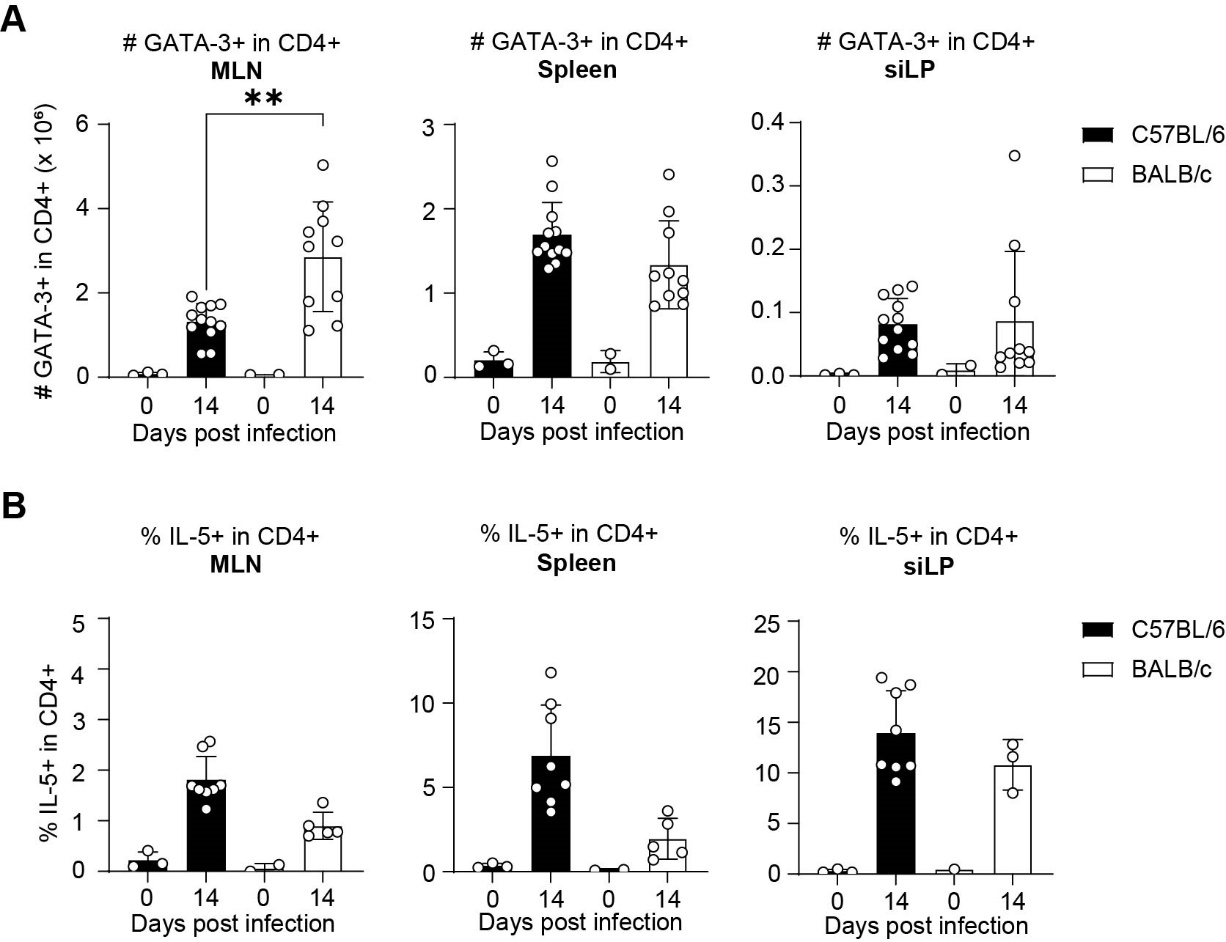
**

**Supplementary Figure 1. Absolute numbers of Th2 cells and IL-5 proportion.** (A) The absolute numbers of GATA-3+ cells in CD4+ T cells from MLN, spleen, and siLP at day 14 post-infection. (B) Frequencies of IL-5+ cells in CD4+ T cells from MLN, spleen, and siLP at day 14 post-infection. Data are pooled from 2-4 independent experiments. Statistically significant differences are indicated; **P*<0.05, ***P*<0.01, Kruskal-Wallis test.

**
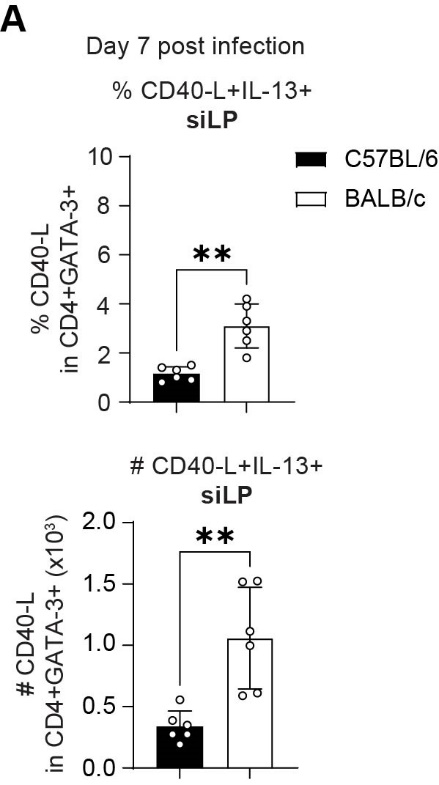
**

**Supplementary Figure 2. Proportions and absolute numbers of parasite-specific CD40-L+IL-13+ cells in GATA-3+ cells in small intestinal lamina propria on day 7 post-infection.** Data are pooled from 2 independent experiments. Statistically significant differences are indicated; ***P*<0.01, Mann-Whitney test.

**
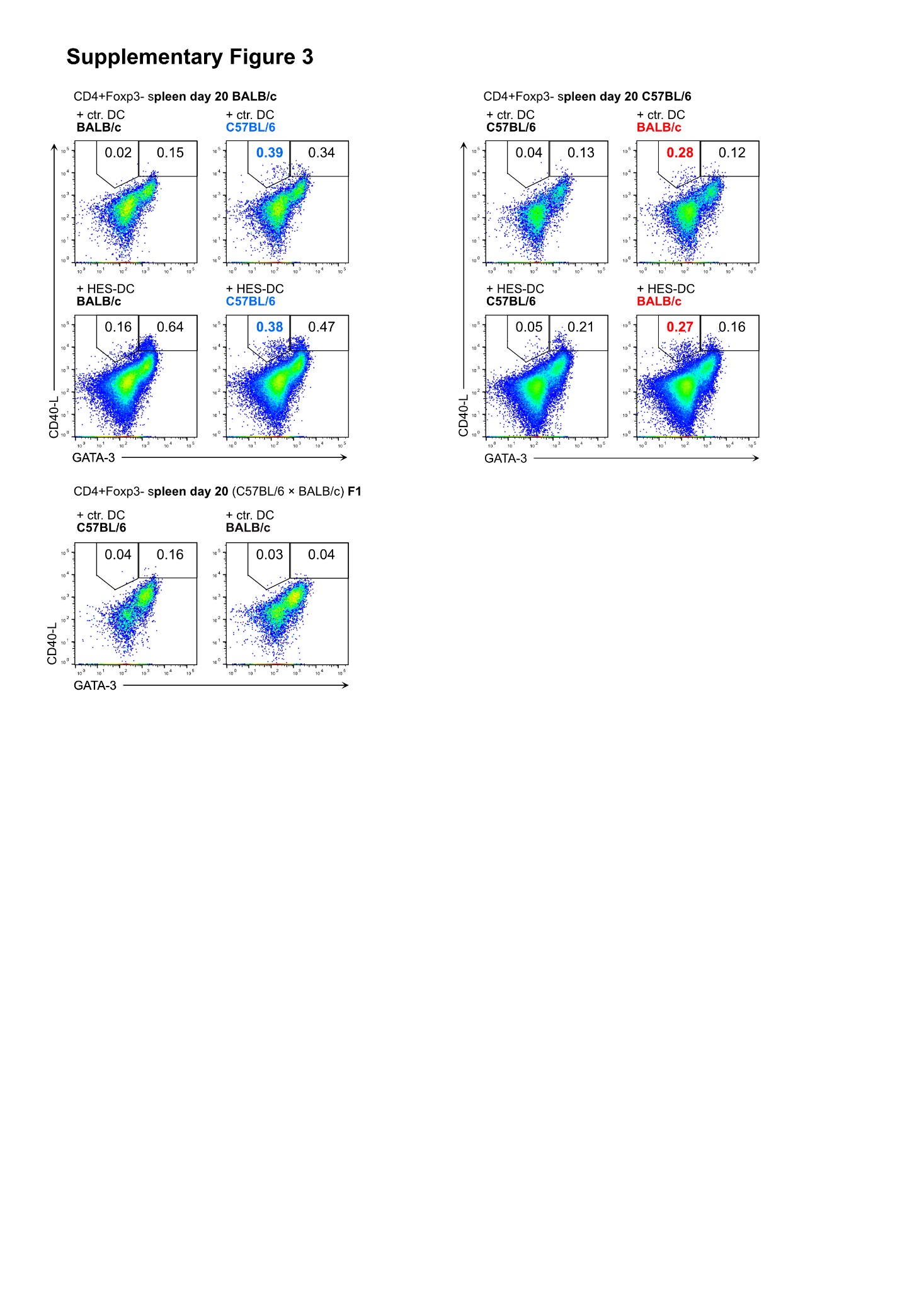
**

**Supplementary Figure 3. CD40-L upregulation by alloreactive CD4+ T cells in coculture with haplotype-mismatched DC.** CD4+ T cells derived from the spleen of day 20 infected BALB/c (left) and C57BL/6 (right) were cultured with matched (1^st^ and 3^rd^ column) or mismatched (2^nd^ and 4^th^ column) bmDC. CD40-L upregulation is seen irrespective of HES loading in plots depicting mismatched cultures (marked by colored gate frequencies). The plots in the third row show the expected absence of alloreactivity in (C57BL/6xBALB/c) F1-derived cell culture with DC from both parental lines.

**
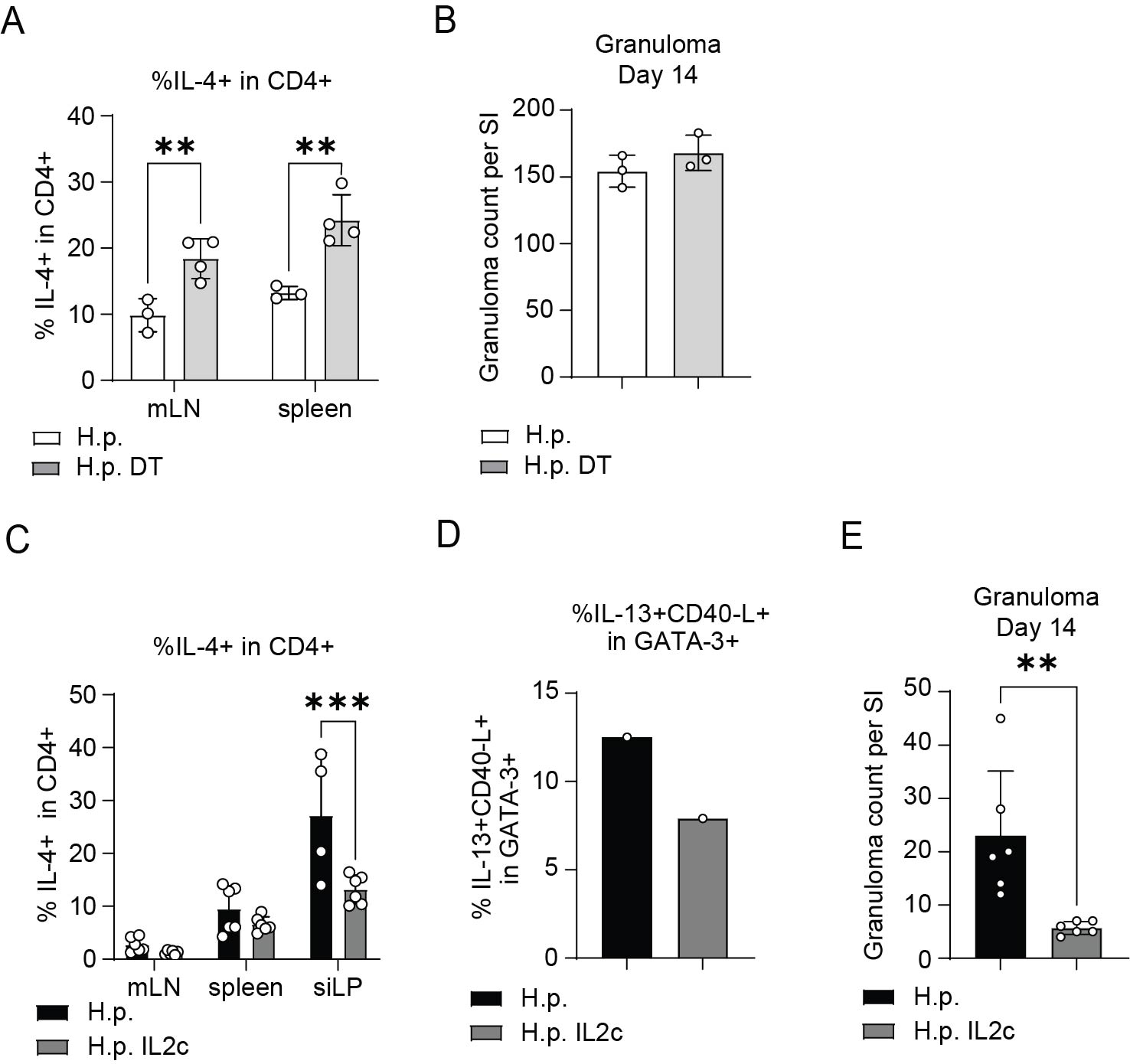
**

**Supplementary Figure 4. Divergent outcome of Treg depletion/expansion in *H. p. bakeri*-infected BALB/c and C57BL/6 mice.** (**A**) Frequencies of IL-4+ cells in CD4+ T cells as determined in MLN, spleen and siLP from BALB/c mice. (**B**) Granuloma count on day 14 from BALB/c mice. (**C**) Frequencies of IL-4+ cells in CD4+ T cells as determined in MLN, spleen, and siLP from C57BL/6 mice. (**D**) Frequencies of CD40-L+ IL-13+ in GATA-3+ cells, as determined in MLN, were generated from pooled MLN samples from 3 C57BL/6 mice. (**E**) Granuloma count on day 14 from C57BL/6 mice. Data are pooled from 2 independent experiments, with n=2-3 mice per group. Statistically significant differences are indicated; ***P*<0.01; ****P*<0.001, Mann-Whitney or Kruskal-Wallis test.


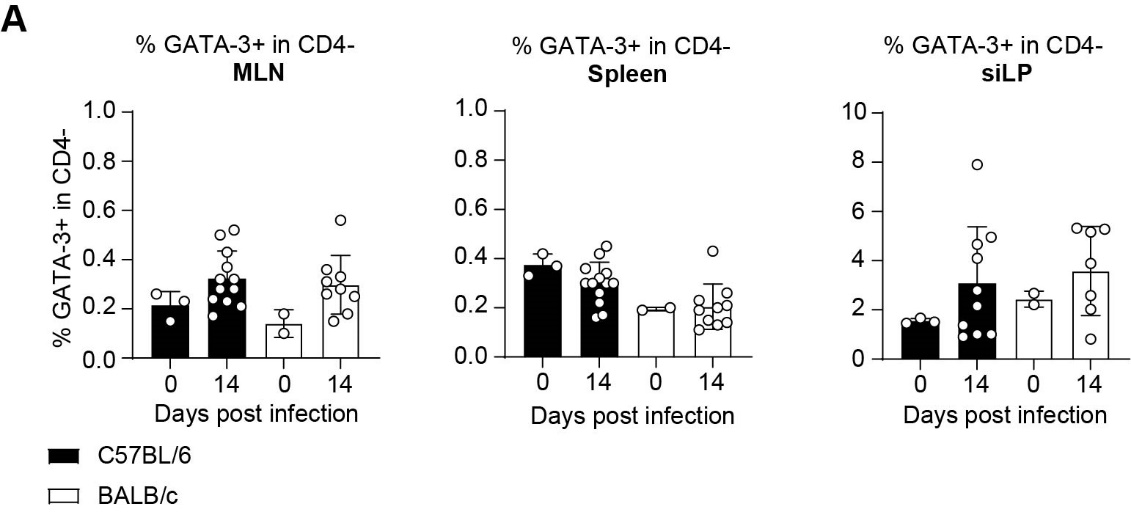


**Supplementary Figure 5. Frequencies of CD4-GATA-3^high^ ILC2 -like cells.** (A) Frequencies of GATA-3⁺ cells within the CD4- cell population in the mesenteric lymph nodes (MLN), spleen, and small intestinal lamina propria (siLP) at days 0 and 14 post-infection. Data are pooled from 3-4 independent experiments, with n=2-4 mice per group. Statistically significant differences are indicated; Kruskal-Wallis test.
